# Phenotypic Nonspecificity as the Result of Limited Specificity of Transcription Factor Function

**DOI:** 10.1155/2018/7089109

**Published:** 2018-10-28

**Authors:** Anthony Percival-Smith

**Affiliations:** Department of Biology, The University of Western Ontario, London, ON, Canada N6A 1B7

## Abstract

Drosophila transcription factor (TF) function is phenotypically nonspecific. Phenotypic nonspecificity is defined as one phenotype being induced or rescued by multiple TFs. To explain this unexpected result, a hypothetical world of limited specificity is explored where all TFs have unique random distributions along the genome due to low information content of DNA sequence recognition and somewhat promiscuous cooperative interactions with other TFs. Transcription is an emergent property of these two conditions. From this model, explicit predictions are made. First, many more cases of TF nonspecificity are expected when examined. Second, the genetic analysis of regulatory sequences should uncover* cis*-element bypass and, third, genetic analysis of TF function should generally uncover differential pleiotropy. In addition, limited specificity provides evolutionary opportunity and explains the inefficiency of expression analysis in identifying genes required for biological processes.

## 1. Introduction: The Specific World of TFs

In Biology gene expression is a major concern and is focused on how a single genotype exhibits multiple phenotypes. The classical example is an* E. coli* cell grown in glucose has very low levels of beta-galactosidase activity, whereas, the same cell will have high levels when grown in lactose [1]. Developmental biology provides nice examples of cells with the same DNA sequence, or genotype, giving rise to specialized transparent lens cells and red blood cells packed with hemoglobin. These distinct phenotypes are the result of differential programs of gene expression. The regulation of gene expression occurs at many different points along the flow of genetic information. One major mechanism of regulation is the control of the initiation of transcription, which is mediated by TFs. Eukaryotic TFs are generally thought to be composed of multiple functional domains: a DNA binding domain that recognizes a specific DNA sequence, a transcriptional regulation domain, and allosteric regulation domain(s) [2, 3]. This multipartite organization confers multiple levels for the regulation of TF specificity. Mechanisms of specificity include specific DNA sequence recognition mediated by the DNA binding domain and regulation of the activity of the TF prior to binding DNA or after binding DNA. In this paper, I will assume that the initial binding of TFs sets in motion the recruitment of RNA polymerase and all subsequent gene and chromatin modifications and, therefore, although important, these epigenetic mechanisms are not discussed.

**Figure 1:**
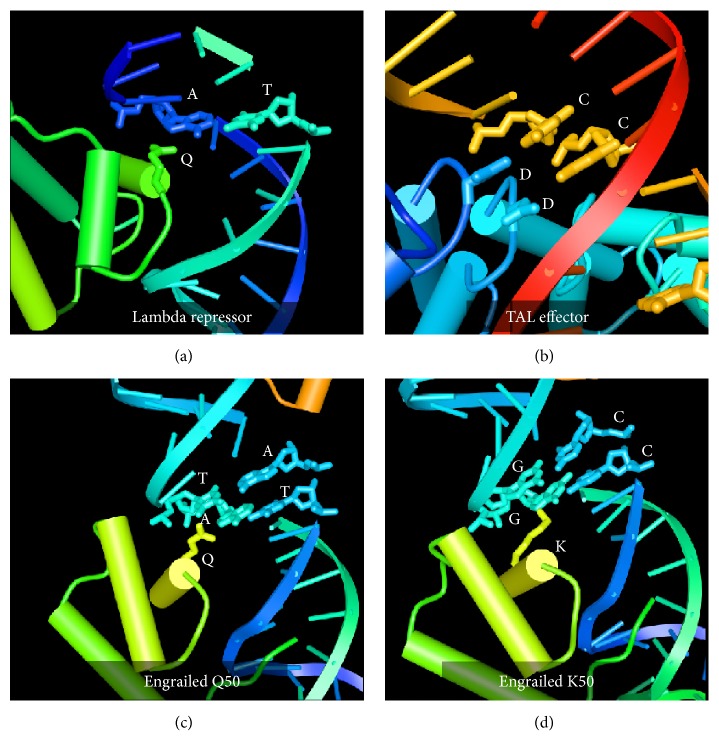
The specific amino acid base interactions of DNA binding protein domains with their recognition site. The interaction of a glutamine (green) of the 434 lambda repressor with a specific A:T base pair (blue:teal) (a) [4]. The interaction of the aspartic acids (blue) of two HD TAL repeats with cytosine bases (brown) (b) [5]. The interaction of the glutamine (yellow) of the wild-type Engrailed Q50 homeodomain with TA:AT base pairs (teal:blue) (c). The interaction of the lysine (yellow) of the change of specificity mutant Engrailed K50 homeodomain with GG:CC base pairs (teal:blue) (d) [12]. All images were generated with the Cn3D rendering program from the coordinates in the NCBI database [13].

The first mechanism regulating TF specificity is specific DNA sequence recognition by the DNA binding domain. The evidence for this mechanism is reflected in the thousand or so structures of protein DNA complexes solved to date. Visualizing amino acid base interactions of a protein DNA complex was a watershed moment in the study of gene expression [4]. From the first structures of lambda repressors to the structure of many more protein DNA complexes, clear, specific amino acid base interactions were observed (Figure 1(a)). Perhaps the most beautiful mechanism of specific DNA recognition is the plant virulence TFs, TAL effectors [5]. The DNA binding domain of TAL TFs is a series of repeats of 34 amino acids with each repeat recognizing a specific base (Figure 1(b)). This one to one repeat to base recognition allows the engineering of proteins that can specifically recognize any DNA sequence in a genome [6]. Indeed, the idea that specific amino acid base contacts were made in the protein DNA complex leads to the isolation or design of change of specificity mutants [7–11]. The structural basis for a change of specificity in a homeodomain (HD) has been determined [12] (Figures 1(c) and 1(d)). The studies of change of specificity mutants reinforce the idea that there are mechanisms of specificity required for the regulation of gene expression. Zooming out from the specific interactions at the interface of a TF DNA complex reveals the TF DNA complex on the promoter regulating the expression of a gene. Important for genetic analysis of gene expression, mutations in the DNA recognition site result in misregulation of gene expression identifying the sequence as a* cis*-acting regulatory element.

Although the structural studies beautifully illustrate how TFs recognize specific DNA sequences, there are long-standing questions about how TFs find the specific targets in complex genomes. Mathematical analysis of the kinetics of the lac repressor operator interaction in the* E. coli* was a major topic 40 years ago. One hypothesis proposed for the lac repressor system is that the lac repressor slides on one-dimensional DNA to find the recognition site, and this hypothesis is supported by image analysis in living cells [16]. In addition, analysis of the binding constants and kinetics of the protein DNA interactions point out two interesting properties. First the difference between the dissociation constant (K_d_) for recognition of specific DNA sequences and the nonspecific sequences can be large in the case of the lac repressor (10^−7^-10^−3^) or small in the case of the HD (10^−2^) [17, 18]. In cases of small differences, it does not take much nonspecific DNA to compete for binding with the recognition site. Second, for a dynamic system the dissociation constant needs to be large enough such that the TF dissociates from the DNA recognition site; otherwise, the TF is bound irreversibly. The half-life of the lac repressor DNA and HD DNA complexes are in the order of 20-30 minutes [18, 19]. In order to have high specificity for binding site recognition and a dynamic system, weak interactions between TFs in prokaryotes and eukaryotes occur and are a mechanism of cooperativity [20]. In prokaryotes many TFs oligomerize and the complex recognizes a sequence of 6-8 nucleotides and these oligomers interact on DNA using weak interactions to effectively increase the size of the target site recognized to 12-16 nucleotides. This is also observed in eukaryotes with the interactions between the Homeotic selector (HOX) proteins and Extradenticle (EXD) [21]. The cooperative interaction between HOX proteins and EXD increases the target size recognized and confers distinct target site recognition to many HOX proteins when in the complex with EXD. With the addition of Homothorax to the HOX EXD complex the target site becomes larger. However, in vitro analyses identifying the sequence of high affinity sites identifies many sequences that are not identified in the genome as occupied in a ChIP seq analysis [22]. Despite decades of work, how TFs find target sequences is still an interesting and open issue.

The application of information theory to the sequence information recognized by the DNA-binding domain of TFs establishes a clear difference between bacterial and eukaryotic TFs [23]. The DNA binding sites recognized by bacterial transcription factors have higher information content than the bacterial genome to be searched harboring these sequences; whereas, the DNA binding sites recognized by eukaryotic transcription factors have much lower information than the genome being searched and significantly lower information than bacterial TF DNA binding sites. Bacterial TFs recognize specific DNA elements in the genome because the DNA binding sites present enough information for specific binding; whereas, eukaryotic TFs do not recognize enough information in the DNA binding sites resulting in a large amount of spurious binding throughout the genome. This problem with the information present in the interaction between a eukaryotic transcription factor and its DNA binding sites is the heart of the Futility Theorem which asserts that essentially all predicted TF binding sites generated with models for the binding of individual TFs have no functional role [24]. This Futility Theorem still holds today for the analysis of eukaryotic TFs and particularly human TFs [25].

A second mechanism of specificity is the regulation of TF activity. TFs are regulated either prior to the formation of a DNA protein complex or after. During development, a simple mechanism of regulation prior to complex formation is whether the TF is expressed in the specific cell or not. Expression is not the only mechanism of regulation, and other mechanisms that regulate TF factor activity prior to DNA binding include regulation of subcellular localization. Nuclear receptor activity is regulated by whether it is in the nucleus or not, a mechanism shared by the NFkB family of TFs [26, 27]. In addition, TF activity is regulated after the formation of the DNA protein complex. An example of this is the yeast Gal4p transcription factor being bound to the* cis*-regulatory sequences in the presence or absence of galactose [28]. Gal4p activity is controlled by whether it is bound to Gal80p or not. The analysis of the last two mechanisms often involves the genetic dissection of the functional domains of the TF to identify the domains required for specific subcellular localization, binding specific regulatory proteins or allosteric effectors. The best examples of the effects of allosteric effectors on TF function are allolactose on the lac repressor interaction with DNA and steroids on nuclear factors. The specific world of transcription factor function is presently the major model for describing TF function. In the proceeding section, I propose an alternate model for eukaryotic TF function. Although in describing this model, I do not incorporate or discuss much from the specific world; I do acknowledge that there are specific mechanisms regulating TF function, which would have to be incorporated into a more complete model for the regulation of the initiation of transcription of all genes in the genome.

## 2. A World of Limited Specificity

### 2.1. Hypothesis: Limited Specificity of TFs

**Figure 2:**
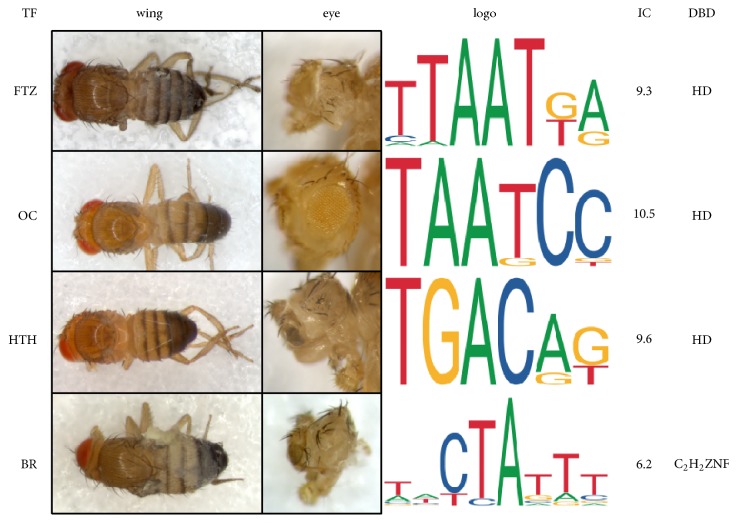
Phenotypic nonspecificity in Drosophila. The transcription factors are Fushi tarazu (FTZ), Ocelliless (OC), Homothorax (HTH), and Broad (BR). Wing and eye refer to the wingless and eyeless phenotypes induced by ectopic expression of the respective TF by either the rhomboid GAL4 or eyeless GAL4 driver [14]. The sequence logos for the recognition sequences of the four TFs are from the JASPAR database [15]. The information content (IC) for the recognition site is listed along with the DNA binding domain (DBD) of the TFs either the homeodomain (HD) or the C2H2 Zn fingers (ZNF).

Extensive phenotypic nonspecificity of TF function has been observed in Drosophila [14, 29–34]. In these observations, phenotypic nonspecificity is defined as one phenotype being induced or rescued by multiple TFs (Figure 2). Phenotypic nonspecificity is observed for HD containing TFs in the induction of wingless, eyeless, arista to tarsus transformations, ectopic thoracic beards,* Hox*-mediated control of autophagy, suppression of* spalt* expression phenotypes following ectopic expression and the rescue of neuromere development and mesoderm formation. This phenotypic nonspecificity was somewhat expected because the HOX DNA binding domain (the HD) recognizes the same recognition sequence. But surprisingly, the phenotypic nonspecificity is not just restricted to HD containing TFs. Both HD containing and non-HD containing TFs when ectopically expressed induce wingless, eyeless, ectopic thoracic beard phenotypes, and the reduced maxillary palp phenotype of* proboscipedia* is rescued by expression of the TF Doublesex male [14]. In the examples of phenotypic nonspecificity (Figure 2), four TFs that induce wingless and eyeless phenotypes have distinct DNA recognition sites of low information content and three are HD TFs and one is a Zn-finger TF. These observations by themselves do not prove a particular mechanism. Here I propose a mechanism and use this model to make explicit testable predictions that would support the hypothesis.

**Figure 3:**
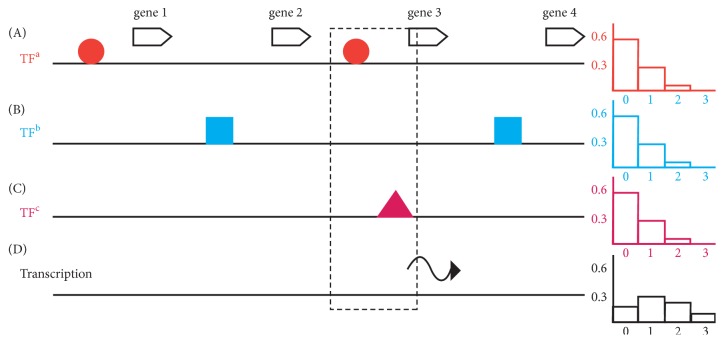
A simplified representation for the nonspecific model for transcriptional regulation. The pattern of binding of three TFs (TFa red circle, TFb blue square, and TFc magenta triangle) to a region of a genome with four genes (A-C) is shown. To the right of each distribution is the expected proportion of genes in the genome with no, 1, 2, and 3 TFs bound. The dotted box indicates a gene with two TFs bound, which in this simplified representation, is sufficient to recruit RNAP for transcription. Transcription (wiggly line) is an emergent property of random TF distributions. To the right is the expected proportion of genes in the genome with no, 1, 2, and 3 of these three TFs bound.

Three different models for TF function can be imagined. A true nonspecific model where every TF has no DNA sequence preference for binding which would lead to an absence of differential gene expression, and an extreme model of specificity where TFs find the promoters of specific sets of genes relying on a high degree of specific DNA sequence recognition which is the combined result of the DNA recognition of binding sites by individual DNA binding domains of TFs and coordinated with very specific protein::protein interactions between only a few TFs for cooperative binding to increase the information content of the DNA protein complex interaction. In the third model, I propose that the DNA sequence recognition and cooperative interactions are limited in specificity and not sufficient to target the expression of a very limited set of genes required for a specific phenotype. This model of limited specificity of TFs is supported by TF occupancy observed in a genome browser (ChIP seq) [35] at two different scales: at the small scale of a few kilobases TFs look to recognize specific regions (TF binding sites) of a promoter, but at a genome scale of megabases each TF is seemingly randomly distributed, and each random distribution of a TF is specific to each individual TF. I propose the distribution of TFs is dependent on the recognition of DNA sequences by the DNA binding domain and cooperative interactions between TFs. The low information content of the DNA sites leads to multiple binding of TFs all along the genome (for a TF that recognizes five bases, this would be a binding site on average every kilobase), and these distributions are refined by cooperative interactions. The cooperative interactions between TFs are also of limited specificity: some TFs interact homophilically, some TFs do not interact homophilically, and all TFs have a very large set of heterophilic interactions.  Figure 3 shows the distribution of three TFs, the proportion of genes that have 0, 1, 2,… TFs conforms to a Poisson distribution. (For simplicity's sake I have assumed each TF recognizes a binding site of the same length, each TF has the same ability to recruit RNA polymerase, and all genes are the same size). Transcription is an emergent property of these random distributions of TFs. To keep it simple in Figure 3, I assume that a gene with two TFs or more is expressed; however, there is not a good estimate of the real number required. Later I will modify and extend the model to explain default repression. The major point is that random distributions of TFs result in large sets of genes being transcribed. In the model of limited specificity, the genes required for completion of a particular process are a small proportion of the genes regulated by the TF and most genes have no role.

Limited specificity explains phenotypic nonspecificity because it allows the substitution of one TF with another. For example, a process that requires the expression of four genes, and the four genes, plus a hundred more, are regulated by TFa. All that is required for phenotypic nonspecificity is that the expression of TFb in place of TFa is able to result in the expression of the four genes required for the process; even though, the total set of regulated genes are quite distinct between the two TFs apart from these four genes.

I predict that there are many more cases of phenotypic nonspecificity. Indeed, there may be cases reported in the literature that have not been recognized as phenotypic nonspecificity. One interesting potential observation of phenotypic nonspecificity is that the initial set of four TFs identified that transform fibroblasts to pluripotent stem cells is not the only set of four TFs that induce pluripotency [36, 37]. Future analysis of the induction of pluripotency may uncover an even larger set of TFs capable of inducing this phenotype. In addition, in an analysis of the neuron cell fates of the Drosophila optic lobe, phenotypic convergence was observed where a neural trait is regulated independently by different combinations of transcription factors [38]. Phenotypic convergence like phenotypic nonspecificity may be a consequence of limited specificity. Because phenotypic nonspecificity is not often tested directly, the following set of predictions is made. First, I predict that many more TF combinations induce pluripotency. Second, the hierarchy of segmentation along the anterior posterior axis of Drosophila uses two large sets of TFs, the Gap proteins and the Pair-rule proteins [39]. Based on my previous observations of frequencies of phenotypic nonspecificity in Drosophila [14], I predict in 30-50% of these genes that the coding region can be substituted by 10-20% of Drosophila TFs and result in normal segmentation. In addition, this pattern of phenotypic rescue will also apply to the set of TFs expressed along the Dorsal ventral axis of the nerve cord in vertebrates, and the set of TFs temporally expressed during the development of the Drosophila brain [40, 41]. Based on the small size of the regulatory regions of yeast genes relative to Drosophila genes, I also predict that 10-20% of yeast transcription factor loci can have their coding regions substituted by 5-10% of yeast transcription factors. If any of these are shown, then these processes require the expression of a set of TFs rather than just the specific set identified, suggesting that TFs do not have intrinsic properties associated with a specific process. Basically, apart from some DNA sequence binding preferences and some preferences for TF partners to interact with cooperatively, TFs are very similar functionally, that is they lack unique and identifying leg, heart or muscle determining protein domains and functions.

### 2.2. A Language Change Associated with Phenotypic Nonspecificity

The language for discussing mechanisms of transcriptional regulation is biased. For example, the formulation of a common question in the study of HOX proteins “Given the high degree of sequence conservation of the HD between HOX proteins and therefore conservation of the DNA binding sites recognized by these HDs, how do the HOX proteins function to specifically direct specific segmental identities during development?” asserts that there is phenotypic specificity of TF function; a mechanism for specificity is assumed and sought at the outset. The question of whether the phenotype regulated by a TF is phenotypically specific or nonspecific is neither discussed nor tested at the outset. The assumption of phenotypic specificity is down to the level of the syntactic articles used. For example, prior to my observation of extensive phenotypic nonspecificity in Drosophila [14], I would have confidently and definitively written that the TF Proboscipedia directs maxillary palp development; however, now I write a TF directs maxillary palp development, because the TF Doublesex Male rescues the proboscipedia phenotype. Phenotypic specificity uses the definite article “the”, and phenotypic nonspecificity uses the indefinite article “a”. In a further example of bias, in the discussion of multiple genes regulated by a TF, the genes are often divided into specific targets and nonspecific, off-targets suggesting that there is an intrinsic difference between these genes regulated by a TF and further implying that off-targets are safe to ignore. This use of language is arbitrarily dismissing a set of regulated genes as unimportant. In the model of limited specificity, more genes regulated by a TF are not required than are required for a process, and therefore, there are no such things as a limited set of confounding off-targets.

### 2.3. Promoter Analysis in a World of Limited Specificity:* cis*-Element Bypass

The mutational analysis of a promoter is a common approach for identifying* cis*-acting regulatory sequences controlling expression of a gene. In a world with limited specificity this approach is still valid, but the interpretation of the results is restricted to an analysis of the present wild-type promoter and not an analysis of the potential of the promoter. When the promoter analysis is being performed, the set of TFs required for regulation of the gene does not change, and hence, does not address the potential of the promoter. If TFa can be substituted for another TFb then the phenomenon of* cis*-element bypass is expected, where the promoter with the mutation in the binding site for TFa is now expressed in the presence of TFb because TFb binds to its own distinct* cis*-element. Conversely, a promoter with a mutation in the* cis*-element binding TFb would be expressed in the presence of TFa, but not when TFa is substituted by TFb.* Cis*-element bypass is an explicit prediction of genetic analysis in the world of limited specificity and would suggest that the* cis*-elements are sufficient but not always necessary. The substitution of one TF for another uncovers the potential of the regulatory region. In a world of limited specificity, arguments about the importance of the organization of* cis*-elements in the promoter for regulation of gene expression are not very important.

### 2.4. Analysis of the Functional Domains of TFs: Differential Pleiotrophy

The aim of mutational analysis of TF loci is determining the functional organization of the proteins by identifying functional domains and motifs important for establishing the wild-type pattern of gene expression. This approach is particularly important in the determination of the modular structure of TFs like Gal4p and nuclear receptors [2, 3]. However, the genetic analysis of other TFs like the Drosophila HD containing TFs Ultrabithorax, Sex combs reduced and Antennapedia, the yeast HD containing TF Pho2p, and Human HOX proteins has uncovered differential pleiotrophy, the nonuniform behavior of mutant alleles across phenotypes suggesting motifs make small tissue specific contributions to overall TF activity [42–48]. The differential pleiotrophy of mutations in TF loci has been attributed to an ensemble nature to allostery of intrinsically disordered proteins [49], but alternatively differential pleiotrophy may also be an expected outcome in the model of limited specificity. In the model, the TF is randomly distributed, and each TF is not much different from another apart from its distribution. The TFs also interact cooperatively with limited specificity resulting in tissue specific TF distributions and activation of transcription. The mechanism of cooperativity is not restricted to two small highly structured protein domains making very specific amino acid interactions with each other such that only a limited number of TF::TF interactions occur. Rather the TF::TF interactions are mediated by more diffuse, nonspecific protein surfaces, and the way the surfaces of TFs interact may change from tissue to tissue and gene to gene. Therefore, the genetic analysis may not identify separate, specific interaction surfaces indicating a high degree of modularity. Rather the diffuse nonspecific surfaces and tissue specific and gene specific deployment of these surfaces show up in genetic analysis as differential pleiotrophy where specific regions of the TF seem to make small tissue specific contributions to overall TF activity. Differential pleiotrophy may be evidence supporting limited specificity.

### 2.5. Phenotypic Nonspecificity as an Evolutionary Opportunity

The study of evolution and development has identified Toolkit genes [50]. Toolkit genes share four major conserved characteristics: structure (basically amino acid sequence), expression, requirement, and function. Many of the Toolkit genes encode TFs. Unfortunately, in the experimental set up testing conservation of function, it is assumed that the function of the proteins is highly specific that is only that specific protein/function can generate or rescue the phenotype. This is particularly true in the analysis of Toolkit TFs. The experimental test is to substitute a TF with an orthologous TF from another organism and determine whether the ortholog can induce or rescue a particular developmental phenotype [51–56]. Observation of phenotypic nonspecificity renders this test uninformative because multiple nonorthologous/nonparalogous TFs can induce or rescue the phenotype [14, 29–34]. Therefore, some toolkit functions are now not functionally conserved, with structurally unrelated TFs able to substitute, severely weakening the Toolkit gene hypothesis at both the levels of conservation of function and structure leaving just conservation of expression and requirement.

In the evolutionary history of animals and plants it is hard to explain how a complex multicellular organism of multiple cell types arises as a result of development. A common assertion is that animals and plants have acquired the genes during evolution to accomplish this feat. A significant proportion of the genes required for determination of the body plan encodes TFs, and hypotheses have proposed that the increase in complexity is associated with the increase in the number of TFs and families of TFs [57]. In a sense, it is proposed that the acquisition of TFs recapitulates phylogeny [58]. This model has two problems. First, phenotypic nonspecificity suggests that TFs are not tailored by evolution to have a restricted role in a specific process. Second, the multiple body plan simplifications observed during evolution confounds the march to complexity. Phylogenomic analysis shows that between divergence of ctenophores and cnidarians, the simple porifera and placozoa phyla branch off; this body plan simplification is also observed in divergence of bilaterian phyla [59–61]. Phenotypic nonspecificity and body plan simplification suggests that a simple addition of genes does not explain or can be used to track the rise of complexity.

Also, an interesting consideration is the effect of the proliferation of transcription factors during evolution in the specific and limited specificity models. In the specific model, a transcription factor like Eyeless/PAX6 becomes associated with the regulation of genes required for eye development and once established is conserved during evolution. The origin of this specificity is difficult to explain. For example, when the gene encoding the TF arose what was the function of the TF at that point? Does it have no function and spends time this way, even though it can bind to DNA and affect transcription, until during evolution it becomes employed for a specific role? Does the TF have an initial function that during evolution is moved to another process and the original role replaced by another TF that has somehow changed its specificity? These are not considerations encountered with limited specificity. From the first time it is expressed, the TF is functional and as long as it does not result in a pattern of gene expression that is detrimental to survival, it is maintained in the genome. During evolution expression of the TF or the genes it regulates may change such at upon genetic analysis in the extant organism it seems to be the essential TF regulator of a process but in reality, it is one of many TFs that have the potential to regulate the process. An expectation of limited specificity is the existence of TF loci that when mutant result in phenotypically viable organisms with no difference in fitness; however, analysis of differential gene expression shows large changes in the pattern of gene expression.

The phenomenon of phenotypic nonspecificity may be good for evolution and development, as phenotypic specificity is an evolutionary constraint, whereas, phenotypic nonspecificity is an evolutionary opportunity. In recently evolved systems involving regulation of multiple genes like the Bicoid and Dorsal morphogens, it would take a large number of random sequence changes in* cis*-elements during evolution in a specific world to bring about the specific pattern of gene expression required. Whereas, in a world of limited specificity, all that is required is a sequence change that results in a change in the expression pattern of a TF that has the potential of bringing about the pattern of gene expression required. Large-scale changes in gene expression can occur in the mechanism of limited specificity with one mutational change because the recognition sites (*cis*-elements) for the TF already exist in the genes required for that phenotypic change. Distalless normally required for limb development can be seconded into a different situation to pattern eye-spots in butterfly wings because the* cis*-elements for Distalless already exist in the genes required for eye spot formation [62]. The set of Dorsal regulated genes, which pattern the early dorsal ventral axis of Drosophila could be a consequence of a simple change in Dorsal expression [63].* Cis*-element bypass uncovers the potential of regulatory regions, which is why it is an important prediction with ramifications for evolution. The world of limited specificity may be a powerful model to explain large-scale changes in gene expression that may have occurred during evolution.

### 2.6. Phenotypic Nonspecificity and the Analysis of Patterns of Gene Expression

In the world of limited specificity, a TF factor is required for the regulation of large sets of genes, the size of which depends on how widely the TF is expressed. In addition, as long as the genes whose products are required for the development of a specific organ are expressed at the correct time, place, and level whether tissue specifically or not is all that matters. These two considerations explain why expression analysis is inefficient at identifying the components required for specific developmental/biological processes. The example I will use is the simple developmental program of sporulation in* S. cerevisiae*, which includes meiosis and the encapsulation of the four haploid products in a spore wall. The general belief in the early 1980s was that genes required for sporulation would be expressed specifically during sporulation; indeed, a few genes specifically expressed during sporulation are required but a large majority of genes specifically expressed during sporulation are not required for sporulation [64–67]. In addition, a systematic screen of genes not required for yeast viability showed that there are more genes required for sporulation that are not specifically expressed during sporulation than genes that are specifically expressed during sporulation, and more surprisingly genes downregulated during sporulation are also required for sporulation [68, 69]. Therefore, identifying genes required for sporulation using an expression approach was inefficient due to two reasons: first a minority of genes specifically expressed during sporulation were required for sporulation, and second the set of genes required for sporulation but not preferentially expressed during sporulation is larger than the set specifically expressed during sporulation and required. This result is expected in a world of limited specificity as the pattern of gene expression is less important than whether the gene is just expressed at the correct time and place irrespective of other considerations and, therefore, questioning the value of preforming expression analysis to screen for genes required for biological processes. And conversely, the expression pattern of a gene may also not provide much information on how it is required. For example, in Drosophila String (Cdc-25) expression is restricted to mitotically dividing cells of the embryo potentially suggesting an important regulatory role for the cell cycle to occur or not. However, expression of String in all cells rescues the string phenotype and does not induce ectopic mitosis, suggesting that String does not have an important role in initiating mitosis [70]. In addition, the TF Fushi tarazu is expressed and required in the even-number parasegments but low-level expression in all cells partially rescues the fushi tarazu phenotype [71]. In the world of limited specificity, the relationships between expression pattern and requirement are not straightforward and do not generally support “guilt by association”. Differential gene expression is a well-described phenomenon; however, limited specificity may change how differential expression is primarily viewed: from a mechanism that promotes the expression of genes that initiate a process to a mechanism that inhibits the expression of genes that might disrupt a process, which would be under selection.

### 2.7. Default Repression

**Figure 4:**
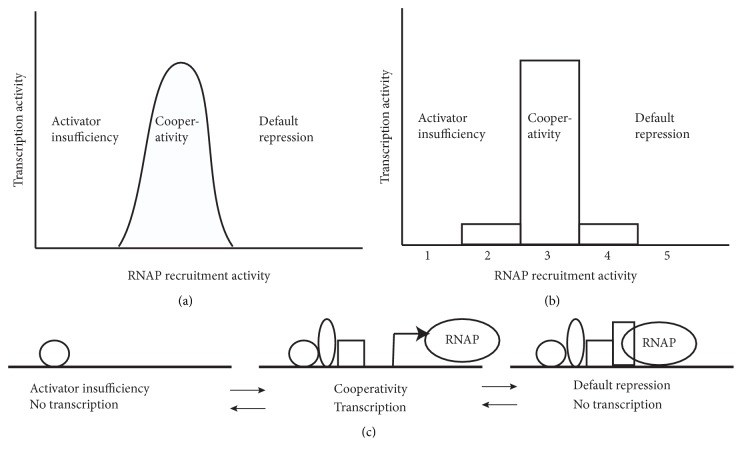
The model for default repression. Transcription activity is the rate of transcription initiation of a gene and RNAP recruitment activity is related to the amount of time RNAP is around the start of transcription of a gene (a and b). In (a) RNAP recruitment activity is assumed to be continuous, but in (b) it is assumed that each TF bound contributes an integer amount of TF recruitment activity that interacts cooperatively to recruit RNAP. It is assumed for simplicity of representation that when three TFs are bound, transcription is at its maximum. The three habits of the gene expression are shown as three interchangeable states (c). When one TF is bound transcription does not occur because of activator insufficiency. When three TFs are bound they cooperate and transcription occurs at the maximum rate. When four TFs are bound RNAP is strongly recruited to the promoter and does not engage in transcription and the gene is in default repression.

When an experimental perturbation is applied, which includes the alteration in the expression of a TF(s), gene expression analysis identifies genes that are both activated and repressed [72]. Interestingly the random distribution of TFs in the genome proposed in the model of limited specificity results in some promoters with few TFs bound and other promoters with many TFs bound. Using this characteristic of the model of limited specificity, I explain the activation/repression phenomenon with the incorporation of the three habits model and poised RNA polymerase II (RNAP) [73–75]. The three habits model explains the behavior of the terminal TFs of major cell-cell signaling pathways. The three habits are: activator insufficiency, where a ligand activated signaling pathway response element binding TFs (SPRE TF) is insufficient for activating transcription of a target gene alone: cooperativity, where a ligand activated SPRE TF with other local activators (TFs) cooperates and activates transcription; and default repression, where in the absence of a ligand the SPRE TF and local TFs repress transcription of the target gene. Although the three habits were proposed to explain observations on the expression of target genes at the end of cell-cell signaling pathways, I will extend it to all genes. I propose that transcriptionally active promoters are in a sweet spot or Goldilocks zone for the number of TFs bound. Starting from no bound TFs (Figure 4), as more TFs bind the amount of RNAP recruitment activity (the term RNAP recruitment is more vague than how it is defined by [76] which is the formation of a closed complex and more reflects time spent around the start of transcription), increase using cooperative interactions and transcription proceeds; however, there can be too much of a good thing where the RNAP recruiting activity is too high and RNAP is conformationally stuck or poised at the promoter. In Figure 4(b), I have shown a simplified example where all TFs have the same recruitment activity. At 1 or 2 TFs bound there is no or little expression (activator insufficiency), at three TFs bound there is the highest level of transcription (cooperativity), but above that number, RNAP is poised on the promoter and less transcription occurs (default repression). This model proposes two mechanisms for the activation of transcription: the gain of RNAP recruitment activity by the addition of TFs and the loss of RNAP recruitment activity by the loss or inactivation of TFs. Most models of transcription put transcription as the end point of RNAP recruitment activity such that poised RNAPs are a prelude to transcription; here poised RNAPs are the consequence of too much RNAP recruitment activity. This explains nicely why in a genome wide analysis of expression, genes are both repressed and activated when a transcription factor is either added (ectopic expression) or taken away (loss of function). When a TF is added many genes now have enough RNAP recruitment activity to be transcribed and, in addition, now other genes have too much RNAP recruitment activity and are not transcribed as highly. Likewise, when a TF is taken away many genes now do not have enough RNAP recruitment activity to be transcribed while other genes have lost enough RNAP recruitment activity to now be transcribed. In this model, the TF is neither a repressor nor an activator, only the number of TFs bound at the promoter sets whether a gene is transcribed or not.

One prediction of this model is the existence of TF loci that reciprocally oscillate between high levels of transcription and high levels of TF accumulation due to both an autoregulatory element with many binding sites for the TF and the TF having a short half-life. The oscillation occurs because at low TF concentration the gene is highly transcribed but as the TF concentration increases and more TFs are bound to the autoregulatory element transcription deceases due to default repression resulting in a subsequent decrease in the concentration of the TF as it is rapidly degraded. New techniques in the live imaging of TF accumulation and active transcription may uncover examples of this [77].

I have no knowledge of experimental examples in eukaryotes of the phenomenon that reduction in RNAP recruitment results in the expression of a gene. However, there may be a bacterial example in the most classical of systems, the lac repressor [78, 79]. The lac repressor interacts with RNAP when not bound to allolactose. The RNAP in this complex with the lac repressor cycles through abortive initiation making short transcripts. The addition of allolactose to the lac repressor RNAP complex results in transient activation of transcription. The exact mechanism of this has been debated and investigated extensively [80]. The heat shock factor and RNAP are bound to (poised) the heat shock promoter in nonheat shocked cells. Poised RNAP II in eukaryotic cells also initiate short transcripts [81]. It is not difficult to imagine that the heat shock response is just temperature sensitive RNAP recruitment, as temperature increases recruitment deceases and transcription is initiated. The model also explains why the mutation of a silencer site results in activation of gene transcription, as the mutation just reduces the number of TFs that bind. In addition, the model explains how a TF can be both an activator and repressor in the same cell as each promoter has a different number of TFs bound. Another prediction of this model is that genes with poised RNA polymerases have more TFs bound than when they are transcribed. To test this prediction, the unbiased detection of the number of TFs bound to a promoter when transcribed or not is the major technical issue that needs to be overcome.

### 2.8. Limited Specificity and the Mathematical Modeling of Gene Expression

One of the interesting aspects of limited specificity is the potential to simplify mathematical modeling of eukaryotic gene expression. In the world of limited specificity all TFs have a similar intrinsic potential, differing only in the relative random distributions of binding in the genome. By applying a few rules to TF behavior, such as the DNA-binding preference and set of interacting TFs, the transcription profile of a cell expressing a defined set of transcription factors could be determined. It is easy to imagine doing this theoretically in a simulation, but it may be possible to take known transcription profiles and TF occupancy profiles to inform such a model and predict the outcome of a perturbation in vivo. The gene expression profile would be represented as a product of a matrix of genes, DNA-binding sites and TFs. Mathematical modeling is made easier in the world of limited specificity because TFs have a smaller number of specific attributes (variables).

## 3. Conclusions

To explain phenotypic nonspecificity of TFs in the induction or rescue of a phenotype, I propose a model where TFs are not inherently much different from one another in terms of intrinsic function and where transcription is an emergent property of the random distributions of these TFs which is influenced by DNA sequence binding preference and somewhat promiscuous TF::TF interactions. Using this model, I make three predictions. First further examination will uncover many more cases of phenotypic nonspecificity. Second, in cases where multiple TFs can function to determine a phenotype,* cis*-element bypass will be observed. To my knowledge, no cases of* cis*-element bypass have been reported because it has yet to be directly tested. Third, genetic analysis of TFs in the nonspecific world will uncover differential pleiotropy. Differential pleiotropy is observed for the HD containing Drosophila, Human and yeast TFs: Ultrabithorax, Fushi tarazu, Sex combs reduced, Antennapedia, HOXA7, HOXB3, and Pho2p; therefore, in order to make a more general statement, observation of differential pleiotropy needs to be extended to other TF families. If the world of limited specificity exists, it has far reaching ramifications for evolution, expression analysis, and mathematical modeling of gene expression.

Although I have been outlining genetic analysis strictly in a world of limited specificity, highly specific mechanisms exist. One example is the specificity conferred to nuclear receptors by the ligand binding domains [82], and another example is the specific and modular interaction between Gal4p with Gal80p [83]. Therefore, if limited specificity exists, the next big problem would be what proportion of the observed patterns of gene expression does it explain? For example, if limited specificity explains 90% of gene expression where specificity only explains 10% then it would have to be considered the dominant model.
